# Dynamics of Intraocular IFN-γ, IL-17 and IL-10-Producing Cell Populations during Relapsing and Monophasic Rat Experimental Autoimmune Uveitis

**DOI:** 10.1371/journal.pone.0049008

**Published:** 2012-11-14

**Authors:** Ulrike Kaufmann, Maria Diedrichs-Möhring, Gerhild Wildner

**Affiliations:** Section of Immunobiology, Dept. of Ophthalmology, Klinikum der Universität, Munich, Germany; University of Southern California, United States of America

## Abstract

A major limitation of most animal models of autoimmune diseases is that they do not reproduce the chronic or relapsing-remitting pattern characteristic of many human autoimmune diseases. This problem has been overcome in our rat models of experimentally induced monophasic or relapsing-remitting autoimmune uveitis (EAU), which depend on the inducing antigen peptides from retinal S-Antigen (monophasic EAU) or interphotoreceptor retinoid-binding protein (relapsing EAU). These models enable us to compare autoreactive and regulatory T cell populations. Intraocular, but not peripheral T cells differ in their cytokine profiles (IFN-γ, IL-17 and IL-10) at distinct time points during monophasic or relapsing EAU. Only intraocular T cells concomitantly produced IFN-γ, IL-17 and/or IL-10. Monophasic EAU presented rising numbers of cells expressing IFN-γ and IL-17 (Th1/Th17) and cells expressing IL-10 or Foxp3. During relapsing uveitis an increase of intraocular IFN-γ+ cells and a concomitant decrease of IL-17+ cells was detected, while IL-10+ populations remained stable. Foxp3+ cells and cells expressing IL-10, even in combination with IFN-γ or IL-17, increased during the resolution of monophasic EAU, suggesting a regulatory role for these T cells. In general, cells producing multiple cytokines increased in monophasic and decreased in relapsing EAU. The distinct appearance of certain intraocular populations with characteristics of regulatory cells points to a differential influence of the ocular environment on T cells that induce acute and monophasic or relapsing disease. Here we provide evidence that different autoantigens can elicit distinct and differently regulated immune responses. IFN-γ, but not IL-17 seems to be the key player in relapsing-remitting uveitis, as shown by increased, synchronized relapses after intraocular application of IFN-γ. We demonstrated dynamic changes of the cytokine pattern during monophasic and relapsing-remitting disease with strongly increasing IL-10 expression in intraocular T cells during monophasic uveitis.

## Introduction

The course of human autoimmune diseases is usually chronic or relapsing-remitting, but so far the mechanisms behind disease recurrences have not been fully elucidated. Most animal models of autoimmune diseases present a monophasic course that does not recur spontaneously, nor can it be re-induced. Thus comparison of immune reactions and regulation in relapsing-remitting and monophasic disease in the same mouse or rat strain is generally not possible and it is difficult to investigate the mechanisms underlying recurrences of autoimmune diseases, which go beyond the mere effect of regulatory T cells [1]. Moreover, since most therapies in animal models have to be applied preemptively [2], it is virtually impossible to determine the effect of therapeutic approaches in an ongoing immune response or on relapses. We have developed a model of spontaneously relapsing-remitting experimental autoimmune uveitis (EAU) in Lewis rats, which is induced by immunization with R14, a peptide from interphotoreceptor retinoid-binding protein (IRBP) in complete Freund's adjuvant (CFA) [1], [3]. In contrast, the same immunization protocol with PDSAg, a peptide from retinal S-Ag, results in a strictly monophasic disease. Similar disease courses are also observed after adoptive transfer of T cells specific for these peptides. These two types of EAU in the same strain of rats enable us to directly compare the immune mechanisms that prevent or permit recurrent intraocular inflammation.

Our previous work on the characterization of T cell lines specific for PDSAg or R14, which induce monophasic or relapsing-remitting disease upon adoptive transfer, has revealed that these T cells have different characteristics [1], [3]. Gene array analysis of R14- and PDSAg-specific T cell lines revealed significant upregulation of genes only in the R14-specific T cell lines. The regulated genes belong to pathways that lie upstream or downstream of IFN-γ, which is the hallmark cytokine of Th1 cells, suggesting that the R14-mediated relapsing disease might result from the effects of Th1 cells. Cytokine analysis of R14- vs. PDSAg-specific T cells indeed revealed an increased IFN-γ secretion as well as a predominance of IFN-γ-producing T cells among R14-specific T cell lines, but we also found IL-17-producing cells. [3] The function of Th17 cells in uveitis is not yet clear, and clinical trials of autoimmune uveitis that target IL-17 are in progress.

The roles of IFN-γ and IL-17 in experimental autoimmune uveitis are contradictory; in general, they were both shown to be pathogenic [4], [5], [6]. No special function has been allocated to cells concomitantly producing cytokines of different T helper types like IFN-γ and IL-17. They are regarded as a transition stage between Th1 and Th17 or vice versa [7]. Regulatory T cells are expected to be responsible for preventing relapses of intraocular inflammation, and thus we have looked for Foxp3- and IL-10-expressing cells in the eyes and peripheral lymph nodes during monophasic and relapsing experimental uveitis. Both cell types have been previously described as regulatory cells [8], [9], [10], [11] in EAU [12], [13]. We found fewer Foxp3+ cells among intraocular lymphocytes than among lymph node cells. In contrast, IL-10-producing cells were detected in much higher numbers in the eyes than in lymph nodes.

In this study, we investigated the intracellular expression of effector and regulatory cytokines in intraocular and peripheral T cells during the course of monophasic and relapsing EAU. We detected differences between the two types of EAU with respect to the cytokine pattern of intraocular T cells. Major differences were found with cells expressing multiple cytokines. During monophasic EAU T cells coexpressing IFN-γ and IL-17 increased, while these populations decreased during the primary course of relapsing disease. Intraocular, but not intraperitoneal injection of IFN-γ after the resolution of R14-induced EAU elicited rather synchronized relapses, underlining the role of IFN-γ for relapsing disease. IL-10-producing cells accumulated in the eyes during monophasic disease, even populations producing IL-10 concomitantly with the inflammatory cytokines IFN-γ and IL-17 were dramatically increasing during the course of monophasic uveitis. That similar numbers of Foxp3-expressing cells were found in both types of EAU indicates that Foxp3 might not be the only factor that is required to prevent relapsing uveitis.

## Materials and Methods

### Animals

Lewis rats were bred in our own colony or purchased from Janvier (France). They were maintained under specific pathogen-free conditions with unlimited access to water and rat chow and used for experiments at the age of 6–8 weeks. All animal experiments were approved by the Review Board of the Regierung von Oberbayern (Permit-Number 55.4-1-54-2531-80-10) and conformed to the ARVO Statement on the Use of Animals in Ophthalmic and Vision Research.

### Induction and scoring of EAU

Animals were immunized subcutaneously into both hind legs with a total volume of 200 µl emulsion containing 25 µg peptide PDSAg (bovine S-Ag aa 342–354) or R14 (human IRBP aa 1169–1191) (Polypeptide Laboratories, France), and CFA, fortified with Mycobacterium tuberculosis strain H37RA (BD Biosciences, Germany) to a final concentration of 2.5 mg/ml. Throughout this paper active immunization is referred to PDSAg-CFA or R14-CFA, respectively. Pertussis toxin or B. pertussis was not used. The time course of disease was determined by daily examination of the anterior part of the eyes (“clinical uveitis”) with an ophthalmoscope. Uveitis was graded as described [14]. In brief, 0.5: enlargement of iris vessels, 1: peripupillar infiltration of leukocytes, 2: pupil covered with fibrin clot, 3: hypopyon, 4: anterior chamber hemorrhage. “Onset of EAU” was defined as a score ≥0.5; “Peak of EAU” was defined as highest score of eyes (≥2) on two consecutive days. “Resolution of EAU” was defined as a decrease of clinical signs. “Remission of EAU” was the period with stable scores ≤0.5 between the resolution of the first attack and the first relapse. “Relapse of EAU” was defined as a score ≥1 following a period of complete absence of all clinical signs of inflammation after the first attack of disease. “Late remission of EAU” was defined as score ≤0.5 after a period (minimum 7 days) of complete absence of all clinical signs of EAU after the first attack of EAU in PDSAg- or after the last relapse in R14-induced EAU.

### MHC class II restriction of T cells

P80 cells (mouse mastocytoma cells) transfected with rat CD80 and either RT1.B or RT1.D as described elsewhere [15] were treated with Mitomycin C (50 µg/ml) for 45 min at 37°C, washed and incubated at a density of 2.5–5×10^4^ cells/96 well (U-bottom tissue culture plate) in RPMI 1640/1% rat serum and peptides (20 µg/ml PDSAg or R14, respectively). PDSAg- or R14-specific rat T cell lines established as described in [16] were added in a 1∶1 ratio, cultured in triplicates for 64–72 h. For the last 18 h cells were pulsed with 1 µCi/well 3H-thymidine. Results are shown as stimulation index (S.I.): mean cpm (peptide)/mean cpm (medium control). Tissue culture medium and supplements were obtained from PAA (Germany).

### Isolation of intraocular and lymph node cells

At different time points during EAU animals were sacrificed, inguinal and popliteal lymph nodes as well as eyes were collected. After removal of the lens, single cell suspensions from the remaining eye tissue were prepared using a 70 µm nylon mesh (Falcon, Germany). The resulting cells were washed twice with RPMI1640.

Single cell suspensions of lymph nodes were prepared with the Gentle MACS Dissociator (Miltenyi, Germany), followed by passing the cells through a 70 µm nylon mesh.

### Cytokine analysis by flow cytometry

Cells were either cultured for three days with 20 µg/ml antigen peptide, 1 µg/ml Con A (BD Biosciences, Germany), medium only or directly used ex vivo. For cultivation with antigen or Con A stimulation no APC were added. Four hours prior to staining cells were incubated in 50 ng/ml PMA, 1 µg/ml ionomycin and 1 µg/ml brefeldin (all from Sigma-Aldrich, Germany). For surface staining mouse anti-rat TCR-αβ antibodies (clone R73, eBioscience, Germany) conjugated with FITC or PE, mouse anti-rat TCR-γδ antibodies (clone V65, BioLegend/Biozol, Germany) conjugated with FITC or PE, were used. For intracellular staining cells were fixed and permeabilized according to the manufacturer's instructions for the rat anti-mouse/rat Foxp3 staining kit (eBioscience, Germany) and subsequently stained with mouse anti-rat IFN-γ antibodies (clone DB-1) conjugated with FITC, PE or Alexa Fluor 647, rat anti-mouse IL-17 antibodies crossreactive with rat IL-17 (clone TC11-18H10.1) conjugated with FITC or PerCP/Cy5.5 (both BioLegend/Biozol, Germany) or mouse anti-rat IL-10 (clone A5-4, BD Biosciences, Germany) conjugated with PE or the respective mouse IgG2b isotype control for anti-IL-10 (clone MCP-11, BioLegend/Biozol, Germany). Mouse anti-rat CD68 conjugated with Alexa Fluor 647 (clone ED1, AbD Serotec, Germany) was used for both, surface and intracellular staining. Foxp3-specific staining was performed with the rat anti-mouse/rat Foxp3 staining kit from eBioscience (Germany); antibodies (clone FJK-16s) were conjugated with FITC or PE. Flow cytometry was performed with a FACS Calibur (BD Bioscience, Germany) and lymphocytes were gated based on forward (FSC) and side scatter (SSC). Data were analyzed with the FlowJo software (TreeStar, USA).

### In vivo administration of IFN-γ

Rats were immunized with R14 in CFA as described, uveitis was determined daily. After resolution of the first course of uveitis 30 U of rat recombinant IFN-γ (Biomol, Germany) in 6 µl sterile saline or 6 µl of saline only were injected into the anterior chamber of both eyes of anesthetized rats with a 30 G needle. Other groups received either 0.5 ml saline or 10^4^ U IFN-γ intraperitoneally.

### Statistical analysis

Statistical analysis was performed using the unpaired two-tailed t test. P<0.05 (*) and p<0.005 (**) were regarded as statistically significant.

## Results

### Expression of IFN-γ and IL-17 by intraocular cells during the course of EAU

**Figure 1:**
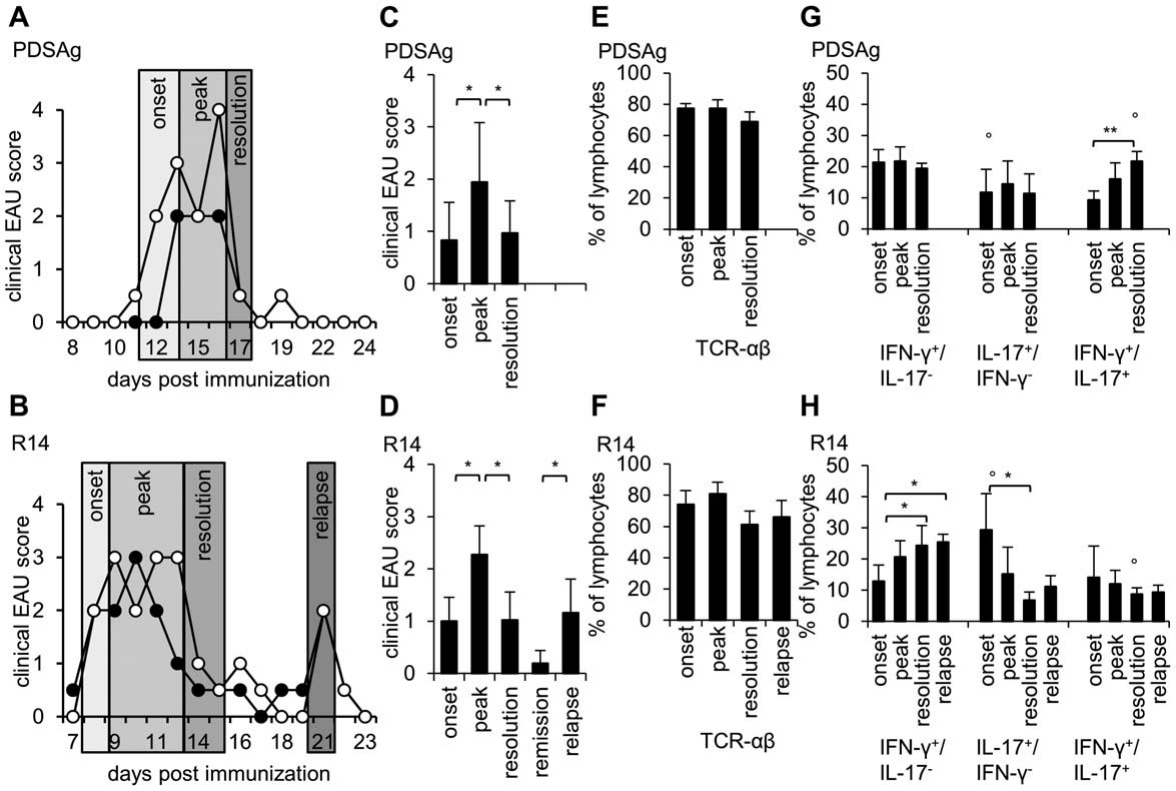
Expression of TCR-αβ, IFN-γ and IL-17 by intraocular cells during EAU. (A, B) Representative clinical courses of monophasic EAU induced with PDSAg (A) or relapsing EAU induced with R14 (B). Black and white symbols represent clinical scores of right and left eyes, respectively. (C, D) Corresponding mean clinical uveitis scores of n = 18–27 eyes from at least 3 independent experiments after immunization with PDSAg-CFA (C) or R14-CFA (D). (E–H) Intraocular cells were isolated from rat eyes at indicated time points after immunization with PDSAg (E, G) or R14 (F, H). Cells from 2–6 eyes were pooled for each experiment and analyzed by flow cytometry. Lymphocytes were gated based on FSC and SSC (see Fig. S1). (E, F) TCR-αβ+ intraocular cells during PDSAg-CFA- (E) and R14-CFA-induced EAU (F). (G, H) Expression of IFN-γ, IL-17 or both together. Data show means from at least 3 independent experiments+SD. Significant differences between the time points of cell collection (*, p<0.05) and PDSAg and R14 (°, p<0.05) are indicated.

**Figure 2:**
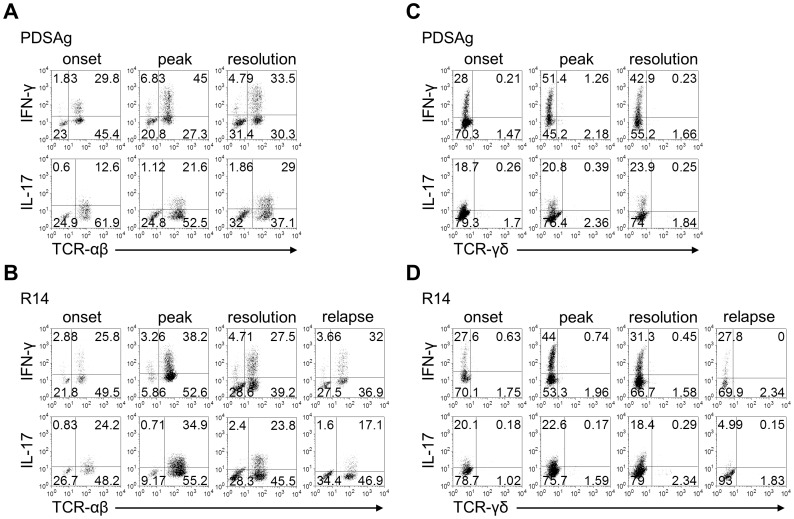
Expression of IFN-γ and IL-17 by intraocular TCR-αβ or TCR-γδ cells. (A, B) Representative dot plots from the FACS analysis of double staining for TCR-αβ and IFN-γ or IL-17 from PDSAg- (A) and R14-induced (B) uveitis. (C, D) Dot plots showing double staining of TCR-γδ and IL-17 or IFN-γ at different time points during PDSAg- (C) and R14-induced EAU (D). Lymphocytes were gated based on FSC and SSC (see Fig. S1). For corresponding EAU scores see Fig. 1C/D.

A panuveitis (inflammation affecting all segments of the eye) was induced by immunization of rats with PDSAg or R14 in CFA. Intraocular cells were isolated during different stages of disease as shown in Fig. 1A/B and C/D, gated for lymphocytes (see Fig. S1) and stained for intracellular cytokines. (Figs. 1 E–H). Immunization with PDSAg-CFA resulted in a monophasic (Fig. 1A), R14-CFA-immunization in relapsing disease (Fig. 1B) according to the clinical uveitis scores. The majority of intraocular lymphocytes were TCR-αβ+ cells, covering 60–80% of the population (Fig. 1E, F). The remaining cells among the “lymphocyte” population are about 2% TCR-γδ+ cells (Fig. 2), about 2–8% CD161+ NK cells (data not shown) and presumably B cells (not tested). We cannot exclude cells of ocular tissues from the population gated as lymphocytes. Only about 1% of CD68+ monocytes/macrophages were detected among the lymphocyte-gated cells (Fig. S1), while the population gated as “macrophages” by forward vs. side scatter still included about 3% TCR-αβ+ cells (Fig. S1B). The macrophage-gated population did not include Foxp3+ or IL-10+ cells and only very few cells expressing IFN-γ or IL-17 (data not shown).

Intracellular staining of intraocular cells from PDSAg-induced EAU, gated for lymphocytes, revealed stable populations of IFN-γ+/IL-17− and IL-17+/IFN-γ− cells during onset, peak and resolution of disease, while the IFN-γ/IL-17 coexpressing cells significantly increased during the course of EAU and doubled from onset to resolution (Fig. 1G). In contrast, during R14-induced EAU the IFN-γ/IL-17 double positive population remained stable, while the numbers of IL-17+/IFN-γ− cells significantly decreased and the IFN-γ+/IL-17− cells significantly increased from onset to resolution and from onset to relapses. The IL-17+/IFN-γ− population was high at onset of R14-induced EAU, then decreased and slightly increased again during the relapse (Fig. 1H). The number of IL-17+/IFN-γ− cells at onset of EAU was significantly higher in R14- compared to PDSAg-induced uveitis (p≤0.05). The major difference between the monophasic and the relapsing disease was the IFN-γ+/IL-17+ population, which was significantly lower in eyes of relapsing, R14- (Fig. 1H) compared to monophasic, PDSAg-mediated EAU (p<0.001, Fig. 1G) during resolution of EAU. Representative flow cytometry dot plots are shown in Fig. S1. The higher percentage of intraocular Th1/Th17 cells during the resolution of the monophasic disease suggests a potential regulatory function of these cells.

### IFN-γ and IL-17 expression by intraocular TCR-αβ and TCR-γδ cells

To better characterize the cells producing IFN-γ and IL-17 we concomitantly stained intraocular cells for TCR-αβ (Fig. 2A, B) or TCR-γδ (Fig. 2C, D). The majority of cells recovered from eyes with EAU were TCR-αβ positive and the population remained rather constant during the course of disease (see also Fig. 1E, F). While IL-17 was almost exclusively produced by intraocular TCR-αβ+ cells in both types of EAU, small numbers of IFN-γ+/TCR-αβ- cells were detected (Fig. 2A, B), but these cells did not bear γδ T cell receptors (Fig. 2C, D).

TCR-γδ+ cells were found in very low numbers (about 2% compared to 60–80% TCR-αβ+ cells) in eyes with EAU and represent only a minor population not exceeding 3.4% of the entire intraocular lymphocyte population. TCR-γδ+ cells did neither produce IFN-γ nor IL-17 (Figs. 2C, D).

### Expression of IFN-γ and IL-17 by intraocular and lymph node T cells after in vitro-stimulation

**Figure 3:**
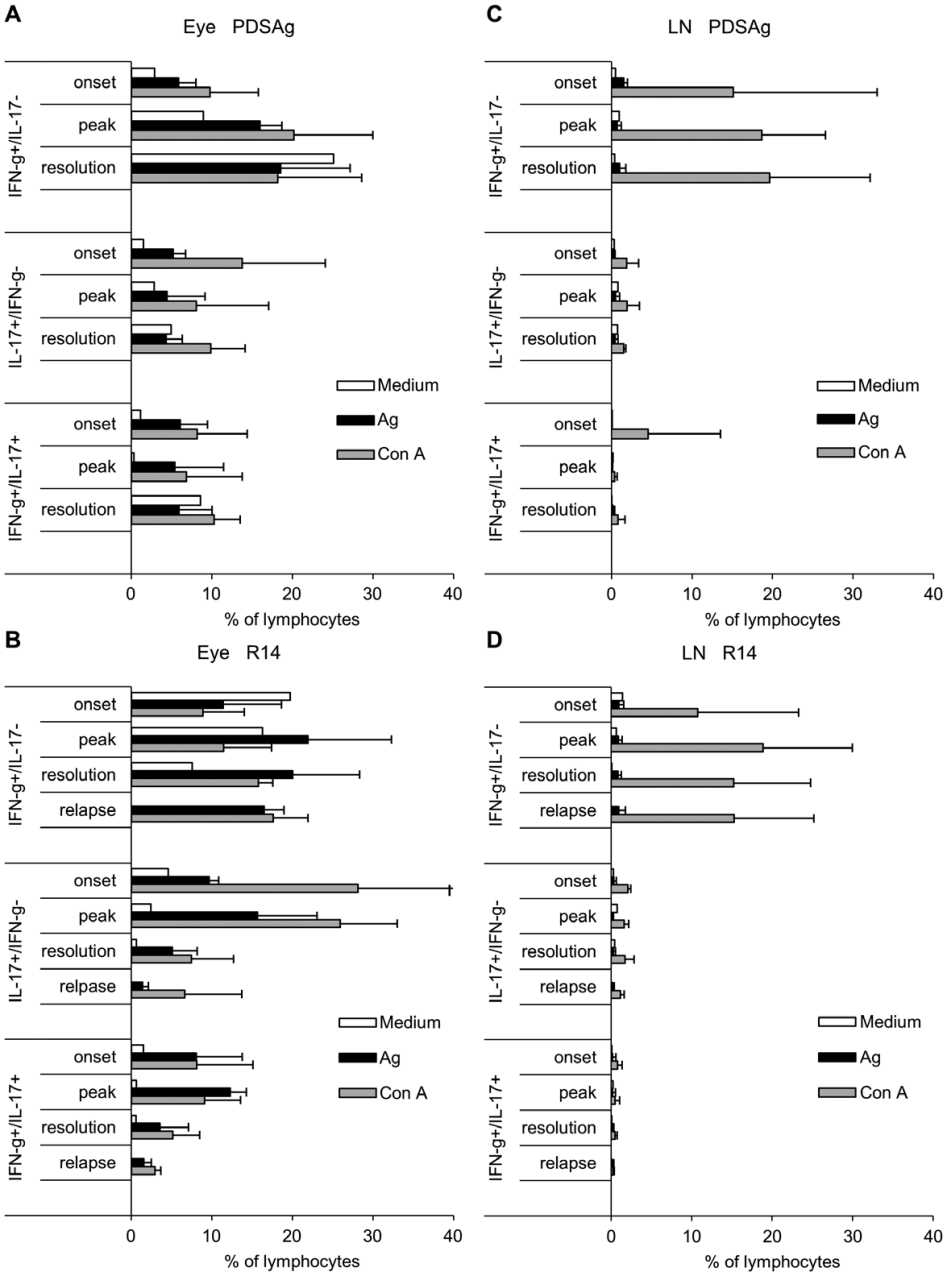
Cytokine expression after in vitro stimulation with antigen or Con A. (A and B) Staining of intraocular cells for IFN-γ and IL-17 during EAU induced with PDSAg-CFA (A) or R14-CFA (B) after 3 days of in vitro incubation in medium only, respective antigen or Con A. C and D show respective data from lymph node cells of the same experiments. Data show means of up to 3 independent experiments (n = 6–18 eyes or lymph node cells from 3–9 animals)+SD. Lymphocytes were gated based on FSC and SSC (see Fig. S1). Corresponding EAU scores are shown in Fig. 1C/D.

In order to determine whether cytokines are produced mainly by autoantigen- or non-autoantigen-specific intraocular T cells, cells isolated from rat eyes were stimulated in vitro for 3 days with either their respective antigen peptide (selecting for antigen-specific T cells) or Con A (polyclonal stimulation) or cultivated in medium only. In PDSAg-induced EAU the number of IFN-γ+/IL-17− cells increased from onset to peak after antigen-stimulation, while the other populations (IL-17+/IFN-γ− or IFN-γ+/IL-17+) remained unaltered and below 10% of intraocular lymphocytes (Fig. 3A). Compared to the medium control, the cytokine-producing, PDSAg- or Con A-stimulated ocular cell populations decreased at resolution, probably representing exhausted T cells. In contrast, antigen- and Con A-stimulated cells from eyes of R14-induced EAU (Fig. 2B) increased with respect to IFN-γ production, while IL-17+ as well as populations producing both, IL-17 and IFN-γ, showed a bell-shaped curve from onset to resolution (Fig. 3B). The pattern of the Con A-stimulated intraocular cell populations from R14-induced EAU (Fig. 3B) was similar to that observed after ex vivo-staining (Fig. 1H).

Intraocular IFN-γ-producing cells responded better to antigen stimulation in vitro than those cells producing IL-17. The latter were better stimulated with the mitogen Con A (Fig. 3A, B).

To address the question whether the pattern of Th1, Th17 and Th1/Th17 cells observed among intraocular T cell populations is reflected by cells from draining lymph nodes of the same rats we isolated popliteal and inguinal lymph nodes, draining the sites of immunization, at the same time points as the respective eyes were examined, and stained them ex vivo and after incubation in medium, with antigen or Con A. Figs. 3C and D show that only Con A-, but not antigen-stimulation induced IFN-γ production in lymph node cells from PDSAg- and R14-immunized rats (Fig. 3C and D). This low frequency of autoantigen-specific T cells might indicate that these cells have already left the draining lymph nodes for the circulation and/or have infiltrated the eyes (as seen in Fig. 3A and B). The lymph node cells might respond with proliferation rather than with cytokine production, which was not determined in this setup. Antigen-specificity of intraocular cells could not be clearly defined with this experimental setup since antigen carried over from eye tissue cannot be completely excluded.

### Expression of Foxp3 by intraocular T cells during the course of EAU

**Figure 4:**
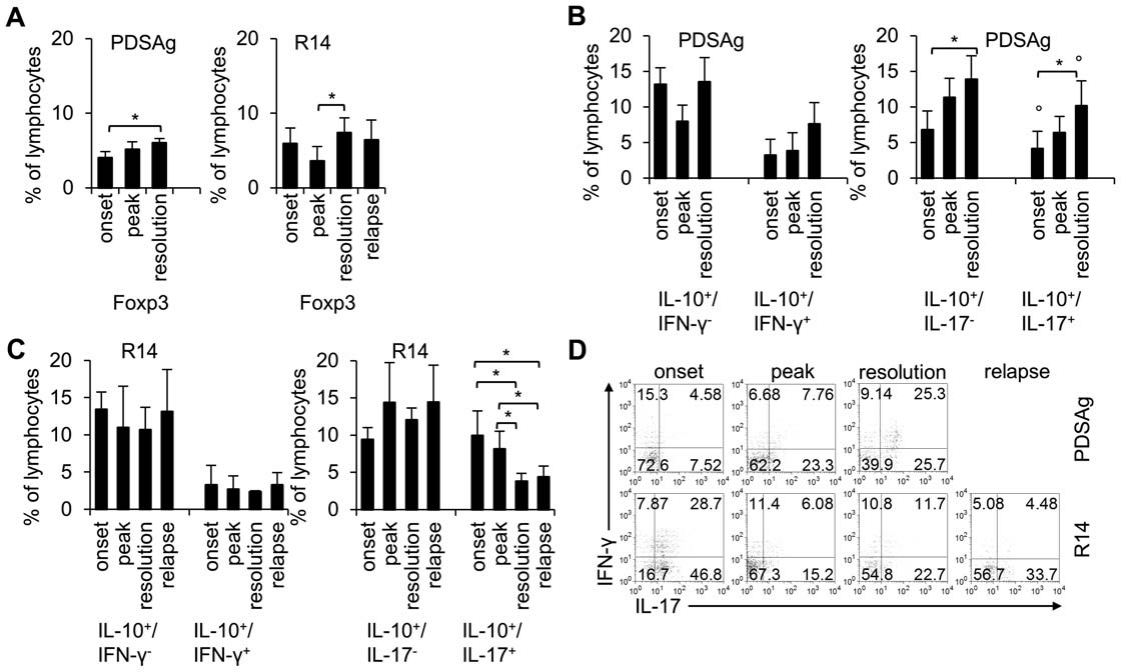
Expression of Foxp3 and IL-10 by intraocular cells during EAU. Cells were isolated from eyes at indicated time points during EAU. Corresponding clinical EAU scores are shown in Fig. 1C and D. Lymphocytes were gated based on FSC and SSC (see Fig. S1). (A) Ex vivo staining for Foxp3. Mean % of cells+SD is shown for at least 3 independent experiments with n = 2–6 eyes per group. (B, C) Ex vivo staining for intracellular IL-10 and IFN-γ expression (left panels) as well as for expression of IL-10 and IL-17 (right panels). Data are shown as mean+SD for at least 3 independent experiments with n = 2–6 eyes per group. Significant differences between the time points of cell collection (*, p<0.05) and PDSAg and R14 are indicated (°, p<0.05). (D) Quadruple staining of intraocular cells for expression of TCR-αβ, IFN-γ, IL-17 and IL-10. Dot plots show IFN-γ and IL-17 staining of TCR-αβ+/IL-10+ gated cells.

The lack of relapses in PDSAg-induced uveitis might be due to the effects of regulatory T cells. We therefore looked for the expression of Foxp3 by intraocular lymphocytes during EAU induced with PDSAg or R14 (Fig. 4A). Although Foxp3+ cells significantly increased from onset to resolution in PDSAg-induced EAU and from peak to resolution in R14-induced disease, there was no significant difference with respect to the number of Foxp3+ cells in eyes (Fig. 4A) or lymph nodes (data not shown) of monophasic or relapsing EAU. We could not detect concomitant expression of Foxp3 with IFN-γ or IL-17, nor with IL-10 (Fig. S2).

Since soluble MHC-class II molecules to define TCR-peptide-recognition are lacking in the rat system, we tried to identify the antigen-specificity of Foxp3+ T cells by in vitro-stimulation of intraocular cells. T cells were incubated with medium only, with their respective antigen or with Con A for 3 days and subsequently stained for TCR-αβ and Foxp3 expression (for representative dot plot see Fig. S3). We observed a 1.4fold increase in Foxp3+/TCR-αβ+ cells after antigen-stimulation and a twofold increase after Con A-treatment with intraocular cells of PDSAg-induced EAU. Intraocular Foxp3+/TCR-αβ+ cells of R14-induced EAU increased 3.6fold after R14- and 4.3fold after Con A-stimulation, indicating a weaker antigen-specific response of Foxp3+ cells in the monophasic type of uveitis that is thought to be less stringently regulated. Nevertheless, since activated rat effector T cells also express Foxp3 (as we have previously published [3]) we still cannot distinguish between regulatory or effector T cells among this population, irrespective of their antigen-specific proliferation.

### Coexpression of IL-10 with IFN-γ and IL-17 by intraocular cells

In addition to Foxp3 expression we determined IL-10 production by intraocular cells (lymphocyte gated) isolated during monophasic (PDSAg, Fig. 4B) or relapsing EAU (R14, Fig. 4C). During PDSAg-induced EAU, cells producing both IL-10 and IFN-γ, slightly increased and IL-10+/IL-17− and IL-10+/IL-17+ populations (Fig. 4B) even significantly increased from onset to resolution (p<0.05). In contrast, during R14-induced EAU the IL-10+/IL-17+ population significantly decreased (p<0.05) (Fig. 4C). There was only a minor population of IL-10+/IFN-γ+ cells (below 5%) in the eyes of R14-induced uveitis, which was stable during the course of disease. In the eyes of R14-induced EAU we found significantly more IL-10+/IL-17+ cells than in eyes of PDSAg-induced uveitis at onset (p<0.05), while the opposite was observed during the resolution of disease (p<0.05) (Figs. 4B and C, right panels). It should be noted that the IL-10+/IFN-γ− population could also include IL-17+ cells and the IL-10+/IL-17− population might comprise IFN-γ+ lymphocytes. In contrast to PDSAg-induced EAU, the IL-10+/IFN-γ+ population did not significantly change during R14-mediated disease, while the number of cells coexpressing IL-10 and IL-17 decreased significantly.

While the numbers of IL-10-expressing cells in lymph nodes remained low during both types of EAU (between 1.4 and 4.3%), they dramatically increased in the eyes (between 14 and 22%, Fig. S2, C and D). Only a minor population of IL-10-producing cells concomitantly expressed Foxp3 (about 1% to 1.7%, Fig. S2).

Staining of TCR-αβ+/IL-10+ intraocular cells for concomitant expression of IFN-γ and IL-17 (Fig. 4D) revealed striking differences between PDSAg- and R14-induced EAU. In eyes of PDSAg-induced uveitis (upper panel) the IL-10+ T cells expressing only IL-17 or both, IL-17 and IFN-γ, massively increased from onset to resolution (IL-10+/IFN-γ+/IL-17+: 4.58% to 25.3%, Fig. 4D upper panel), while the opposite effect was observed with IL-10+ T cells from R14-induced disease: high numbers of IL-10+/IFN-γ+/IL-17+ cells at onset (28.7%) dropped to 11.7% at resolution and less than 4.5% during relapse (Fig. 4D, lower panel). We thus conclude that IL-10+ cells might be responsible for preventing relapses in PDSAg-induced uveitis, while the decreasing number of IL-10+ cells in the eyes of R14-induced uveitis might allow recurrent inflammation.

### Cytokine expression by intraocular lymphocytes during remission of EAU

**Figure 5:**
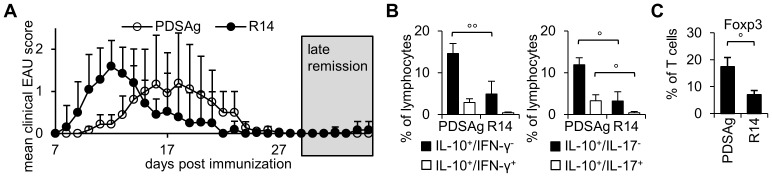
Expression IL-10, IFN-γ, IL-17 and Foxp3 in the late phase of remission of EAU. (A) Time course showing mean daily clinical uveitis scores. Eyes were collected after complete resolution of clinical uveitis (“late remission”) 10 days after resolution of EAU induced as indicated. (B) Coexpression of IFN-γ (left panel) and IL-17 (right panel) with IL-10 in quiescent eyes of late remission. Cells were gated on lymphocytes based on FSC and SSC. (C) Expression of Foxp3 in intraocular TCR-αβ+ cells from quiescent eyes of late remission. Data show means of at least 3 independent experiments with n = 6–10 eyes per group.

We also investigated quiescent eyes (no clinical signs of EAU for 7–13 days) in the late remission of intraocular inflammation (see Fig. 5A) for remaining lymphocytes and their concomitant expression of IL-10 with IFN-γ and IL-17 (Fig. 5B), or Foxp3 (Fig. 5C). PDSAg-induced EAU differed significantly from R14-induced disease (p<0.05) with respect to IL-10+/IFN-γ−, IL-10+/IL-17− and IL-10+/IL-17+ cells (Fig. 5B). Also Foxp3+ T cells were significantly elevated (p<0.05) in eyes during late remission of PDSAg-induced uveitis, indicating the extended presence of elevated numbers of regulatory cells in the eyes of non-relapsing EAU (Fig. 5C).

### MHC class II-restriction of PDSAg- and R14-specific T cell lines

**Figure 6:**
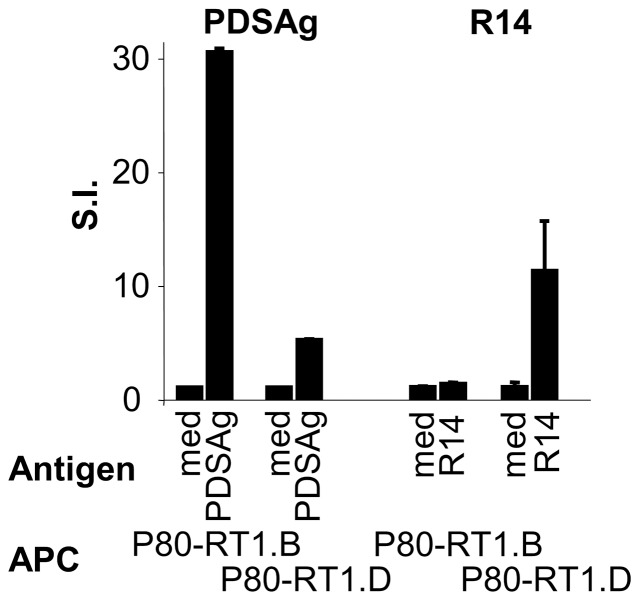
MHC-class II restriction of PDSAg- and R14-specific T cells. Proliferation of a PDSAg- and a R14-specific T cell line in response to the respective antigen peptide and mouse mastocytoma cells P80, expressing rat CD80 and rat MHC-class II antigens RT1.B or RT1.D. T cells were stimulated for 3 days and T cell responses are shown as stimulation index (SI)+SD.

In order to further differentiate between PDSAg- and R14-induced uveitis we investigated the antigen-recognition of the respective T cells. We found that the two antigen peptides, PDSAg and R14, were presented by different MHC class II molecules, as shown by the proliferation of respective T cell lines in response to P80 cells expressing rat CD80 and rat MHC class II RT1.B or RT1.D. R14 was only presented by RT1.D, PDSAg predominantly presented by RT1.B (Fig. 6). The minor stimulation of PDSAg-specific T cells in cocultures with P80-RT1.D and PDSAg indicated a small subpopulation of T cells probably recognizing PDSAg or a degradation product presented by RT1.D.

### In vivo administration of IFN-γ

**Figure 7:**
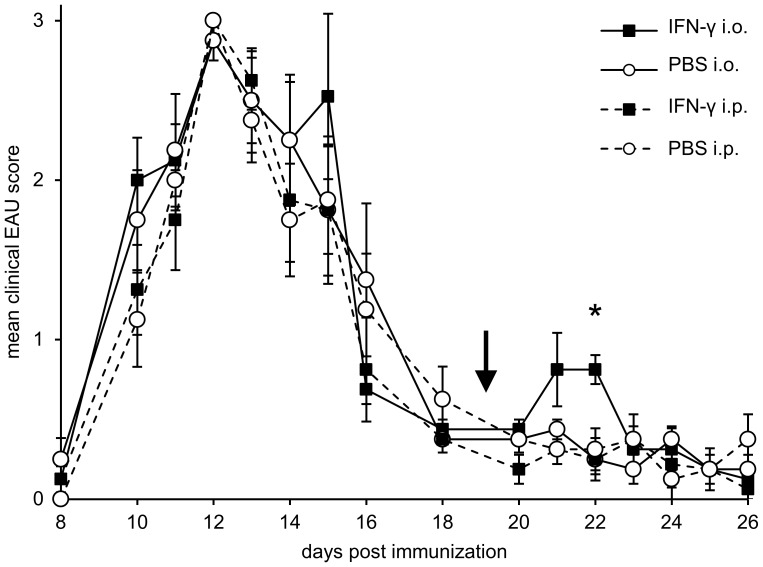
In vivo administration of IFN-γ. Rats immunized with R14-CFA were injected intraocularly (i.o.) into the anterior chamber or intraperitoneally (i.p.) with IFN-γ or saline (as indicated) at day 19, when the clinical signs of EAU were ≤0.5 in all eyes. The time course shows the mean daily clinical scores of all eyes per group ± SE. The arrow marks the time point of IFN-γ or saline application. Significant difference between the group receiving intraocular IFN-γ and the other treatment groups is indicated (*, p≤0.05).

In order to further investigate the role of IFN-γ in relapsing EAU rats received a single injection of IFN-γ into both eyes or intraperitoneally immediately after resolution of the first course of uveitis (Fig. 7). Control groups were treated with similar injections of saline. While spontaneous relapses during R14-induced uveitis appear unpredictable, we observed rather synchronized recurrences of intraocular inflammation with clinical scores of 1–2 in 7 of 8 eyes 3 days after injection of IFN-γ (mean maximum scores of all eyes: 1.0), while only 1/8 eyes injected with saline experienced a relapse (mean score 0.125). Intraperitoneal injection of IFN-γ or saline had no such effect on relapses of EAU, only 1/8 eyes in the groups of intraperitoneal IFN-γ and 4/8 eyes in the group treated with saline i.p. had relapses after 4 to 6 days. These data indicate that relapses of uveitis are initiated by T cells remaining within the eye after uveitis rather than by newly activated T cells invading the eye from the periphery.

## Discussion

A major limitation of most animal models of autoimmune diseases is that they do not reproduce the chronic or relapsing-remitting pattern characteristic of many human autoimmune diseases. They thus do not provide an opportunity to investigate the mechanisms underlying recurrent inflammation. Experimental autoimmune uveitis in Lewis rats can be both a monophasic as well as a relapsing-remitting disease, strictly depending on the peptide used for induction. Therefore we have the unique possibility to investigate two types of EAU in the same rodent strain. EAU induced by S-Ag peptide PDSAg is monophasic, while the IRBP peptide R14 causes relapses of intraocular inflammation. Further differences between PDSAg- and R14-induced diseases are based on the differential regulation of EAU by chemokine variants like Met-RANTES [17] or a mutant of MCP-1/CCL2 [18].

In previous studies we could also demonstrate the differences in the course of EAU after adoptive transfer of T cell lines and differences in gene and protein expression between PDSAg- and R14-specific T cells. We found several genes and signaling pathways upregulated in R14-specific T cell lines, most of them either upstream or downstream of IFN-γ signaling. We also observed differences in IL-17 protein expression between PDSAg- and R14-specific T cell lines. R14-specific T cells were more Th1-prone with enhanced IFN-γ production compared to PDSAg-specific T cells, which expressed more IL-17 [3]. We thus decided to investigate IFN-γ and IL-17 protein expression of intraocular cells from rats during EAU.

The majority of infiltrating lymphocytes in the eye during EAU are T cells expressing αβ T cell receptors. In contrast to a mouse model of EAU [19], we could detect only small numbers of TCR-γδ+ cells within the eyes during EAU, and these cells did not produce pro-inflammatory cytokines such as IFN-γ or IL-17.

In order to separate Th1 from Th17 cells for further experiments, we investigated surface markers of human and mouse IL-17-producing cells such as the NK receptor P1A/CD161 [20], and CCR6, the receptor for CCL20 [21]. Although we could demonstrate the expression of both molecules on rat lymphocytes, none was coexpressed with IL-17 (data not shown), thus neither molecule is suitable for the isolation of IL-17+ cells. Ex vivo-staining of intraocular cells has revealed striking differences between monophasic and relapsing disease with respect to the pattern of IFN-γ+ and IL-17+ cell populations during the course of EAU. Both, IFN-γ- and IL-17-producing populations, which did not coexpress the respective other cytokine, remained quite stable during the course of monophasic EAU (PDSAg), while during the course of relapsing uveitis (R14) IL-17+/IFN-γ− cells decreased and IFN-γ+/IL-17− cells increased. From these observations we conclude that IFN-γ+ cells are responsible for initiating further recurrences in relapsing EAU, while IL-17+ cells might rather have the function to facilitate the primary ocular invasion of cells. IL-17+ cells thus might fulfill different functions in the monophasic compared to the relapsing disease.

Our observations concerning the central role of IFN-γ made by gene array analysis [3] was supported by the induction of synchronized recurrences of uveitis after intraocular injection of IFN-γ. We had previously shown that GFP+ autoreactive T cells remain in the eye for several weeks during remission of EAU, and form clusters at relapses. It seems that these cells have been reactivated by ocular application of IFN-γ, and might have proliferated, which resulted in cluster formation. No effect was observed after intraperitoneal injection of IFN-γ, underlining the pivotal role of intraocular cells during recurrent intraocular inflammation.

Interestingly, we also detected cells concomitantly expressing IFN-γ and IL-17 in the eyes of both types of EAU. In R14-induced relapsing EAU this population slightly decreased during the course of disease, but increased during monophasic, PDSAg-induced uveitis. We speculate that these cells might play a role in prohibiting recurrent inflammation in monophasic EAU, for it was previously shown that injection of IL-17 can ameliorate EAU [22]. A regulatory role of cells coexpressing two inflammatory cytokines seems to be unusual, but further experiments showed that although IFN-γ+/IL-17+ cells did not express Foxp3, a rather high percentage coexpressed IL-10, a cytokine typical for regulatory T cells [23].

Comparing IFN-γ- and IL-17-expressing cell populations from eyes and draining lymph nodes at different stages of uveitis revealed distinct cell populations found exclusively in the affected eyes (especially the IFN-γ+/IL-17+ cells). Only after Con A stimulation we could recover IFN-γ, but no IL-17 producing cells from lymph nodes. Merely a small population of Con A-responsive IFN-γ+/IL-17+ cells was detected among lymph node cells of PDSAg -induced EAU. We thus conclude that the ocular environment has a strong influence on the shaping of invading T cell populations. The availability of autoantigen, which is needed for local reactivation [24] and local cytokine production can influence the intraocular T cell populations. Intraocular cytokines are usually immunosuppressive, leading to the phenomenon of ACAID (anterior chamber-associated immune deviation) [25], while already activated T cells are not hampered by the ocular environment. The intraocular environment seems to have different effects on PDSAg-specific and R14-specific T lymphocytes. With respect to in vitro-stimulation it might as well be possible that the culture conditions favor Th1 subtypes more than other subtypes, resulting in slight differences of T cell populations recovered after in vitro stimulation compared to cells directly stained ex vivo [26]. For instance, it was shown that Th17 cells have an unstable phenotype and can easily switch their cytokine pattern [7], supporting our findings of higher numbers of IFN-γ+ cells after in vitro stimulation.

It is noteworthy that the various stages during the course of EAU (onset, peak, resolution, relapse) reflect the slightly time-delayed consequences of intraocular autoreactive T cell actions. At “onset” of EAU we recovered autoreactive T cells from the eyes that had immigrated and been reactivated some days before, while during “resolution” they might be already downregulated and have stopped recruiting inflammatory cells. What we observe as “clinical uveitis” is characterized by infiltrating inflammatory leukocytes, mainly monocytes/macrophages [24].

We also determined IL-10 expression of intraocular cells that are concomitantly producing IFN-γ and/or IL-17. During the course of monophasic, PDSAg-induced EAU we observed an increase of cells coexpressing IFN-γ as well as IL-17 and IL-10. We do not know whether these populations are immigrating from the circulation during the different stages of uveitis, or whether the T cells are changing their cytokine profiles (and functions?) in the eyes during EAU. During monophasic EAU (PDSAg) the intraocular IL-10+/IFN-γ+ and IL-10+/IL-17+ populations increased, but they were either very small (IL-10+/IFN-γ+) or even decreased (IL-10+/IL-17+) in the eyes of relapsing, R14-induced EAU. Thus we speculate that these cells have a specific function to prevent recurrences of intraocular inflammation after PDSAg-immunization. Since it was shown previously that an effector cytokine like IFN-γ can increase the regulatory effect of IL-10 [27], this effect might also be true for IL-17 in combination with IL-10.

The number of cells producing the suppressive cytokine IL-10 increased during the course of monophasic, PDSAg-specific EAU, while this population decreased during relapsing, R14-induced uveitis. It is not clear how IL-10 production is induced in effector T cells of the Th1 or Th17 type. Jankovic et al. [28] have shown that during the immunization of mice with peptide and CFA the production of IL-6 and TGF-β could induce pathogenic Th17 cells. Further exposure to high concentrations of TGF-β – as is found in the eye – and IL-6, which might be provided by later infiltrating inflammatory cells in EAU, could induce IL-10 production by Th17 cells in the eyes [29], [30]. IL-10 can control both, Th17 [9] as well as Th1 [31] responses. IL-17+ as well as IL-17+/IFN-γ+ cells bearing IL-10 receptors can thus be directly suppressed by extrinsic IL-10 [11]. IL-10, but not Foxp3 production can be induced by IL-27 in IFN-γ-producing cells, which could suppress EAE [8]. The source of IL-27 could be either infiltrating inflammatory cells or local microglia, stimulated with IFN-γ from pathogenic T cells [32]. Concurrent production of IL-10 with inflammatory cytokines like IFN-γ or IL-17 in T cells is also regarded as the “endpoint” of a successful effector response [31] rather than being a distinct T cell lineage. In that case, the increase of IFN-γ+/IL-10+ and IL-17+/IL-10+ cells during PDSAg-induced, monophasic EAU would represent the termination of the T cell response. In contrast, in eyes of R14-induced uveitis these populations were found at constant, very low levels (IFN-γ+/IL-10+) or even decreasing (IL-10+/IL-17+), allowing relapses of inflammation. IL-10 can suppress T cells even intrinsically [33] and might be induced in the T cells already during the first encounter of antigen, depending on the MHC class II restriction. As described by Matsuoka et al. [34] T cells restricted for HLA-DR (which is the human equivalent for the R14-presenting RT1.D in rats) induce more inflammatory cytokines, while DQ-restricted T cells (DQ is equivalent to rat RT1.B, presenting PDSAg) stimulate higher levels of IL-10. Consequently, the life of a T cell is already determined after its first antigen-specific stimulation.

While we recovered only a low number of IL-10+ cells from lymph nodes draining the site of immunization during EAU, we observed an up to tenfold increase of these cells within the eyes. This was in sharp contrast to Foxp3+ cells, which did not differ between lymph nodes and eyes, underlining the important regulatory role of IL-10+ cells within the eye. Since the increase of IL-10+ cells, like IFN-γ and IL-17 coproducing lymphocytes, is only detected in the eyes, we speculate that the IL-10+ regulatory population develops from the autoaggressive T cell pool in the ocular environment, augmented by their own IL-10 production or by IL-10 from retinal cells [35]. This phenotype switch could be a kind of self control of effector cells and has been described for Th1 cells [36], as well as for Th17 cells [9]. The potential of IL-10 to suppress uveitis has been demonstrated by injecting a lentiviral-vector expressing IL-10 into the anterior chamber of the eye [37], by the protective effect of IL-10 injection during the afferent immune response of EAU [13] and by a diminished uveitis in IL-10 transgenic mice [12].

During the course of disease we could detect increasing numbers of Foxp3+ cells in the eyes of PDSAg-induced EAU, but there was no significant difference compared to R14-induced disease. Despite relatively high numbers of Foxp3-expressing cells during resolution and relapses in the eyes of R14-induced EAU we observed recurrent inflammation. Either the Foxp3+ cells found in relapsing EAU have a weaker regulatory function compared to the Foxp3+ cells from monophasic EAU [38], or they were no regulatory T cells at all. In vitro-stimulation of intraocular cells with both, antigen or mitogen, resulted in an increase of Foxp3-expressing T cells, which was higher in cells from R14-induced uveitis. As we have shown previously [3], Foxp3 is also expressed by activated rat effector T cells, an observation that precludes a definite assignment of these cells to the T effector or T regulator population. Interestingly, in quiescent eyes after subsiding of intraocular inflammation, significantly more Foxp3+ cells were found in eyes of PDSAg- compared to R14-induced uveitis, suggesting that these cells might indeed have a regulatory phenotype. Those eyes also revealed significantly increased IL-10+ populations. Thus, we might have Foxp3+ as well as IL-10+ regulatory T cell populations with either distinct or synergistic functions.

Our findings indicate that autoreactive T cell populations can be shaped differently depending on their antigen peptides, followed by the influence of the environment of the target tissue. Investigations of the role of antigen presentation and recognition of T cells are necessary to better understand the development of different T cell characteristics that lead to monophasic or relapsing disease. Furthermore the clear classification of T cells expressing multiple cytokines, either combinations of effector cytokines or of effector and regulatory cytokines, is essential for understanding their role in autoimmunity.

## Supporting Information

Figure S1 **Representative dot plots from staining of intraocular cells.** (A) Gating of the “lymphocyte” population of intraocular cells according to FSC and SSC (left panel) and the respective staining for TCR-αβ and monocytes/macrophages (CD68) (right panel). (B) Gating of the “macrophage” population of intraocular cells by FSC and SSC (left panel) and the respective staining for TCR-αβ and CD68 (right panel). (C) Representative dot plots of IFN-γ and IL-17 staining of the lymphocyte population, gated as shown in Fig. S1A. (D) Staining of intraocular lymphocytes (gated as shown on left panel) for TCR-αβ (versus FSC) and double staining for TCR-αβ and IL-10. The right panel shows double staining with anti-TCR-αβ and the isotype control for the anti-IL-10 antibody.
(TIF)

Figure S2 **Coexpression of Foxp3 with IL-17, IFN-γ and IL-10 by intraocular and lymph node cells.** Representative dot plots of the FACS analysis of “lymphocyte”-gated (see Fig. S1) intraocular (A, B) and lymph node cells (C, D) at different time points of PDSAg- and R14-CFA-induced uveitis. Cells were stained for Foxp3 and coexpression of IL-17 (upper panels), IFN-γ (middle panels) or IL-10 (lower panels). (E) Representative isotype controls for anti-Foxp3, anti-IL-17 and anti-IFN-γ, shown with lymph node cells.
(TIF)

Figure S3 **Foxp3 expression of antigen- and Con A-stimulated intraocular cells.** Representative dot plots of cells from the eyes during resolution of PDSAg- and R14-induced EAU, stained for TCR-αβ and Foxp3 after 3 days of culture in medium only, specific antigen or Con A (without addition of APC). Cells were gated for lymphocytes according to SSC and FSC.
(TIF)
