# Supplementary figures and images for: Dynamics of Intraocular IFN-γ, IL-17 and IL-10-Producing Cell Populations during Relapsing and Monophasic Rat Experimental Autoimmune Uveitis
Source: PLoS One. 2012 Nov 14;7(11):e49008. doi: 10.1371/journal.pone.0049008 (PMC3498374; doi:10.1371/journal.pone.0049008)

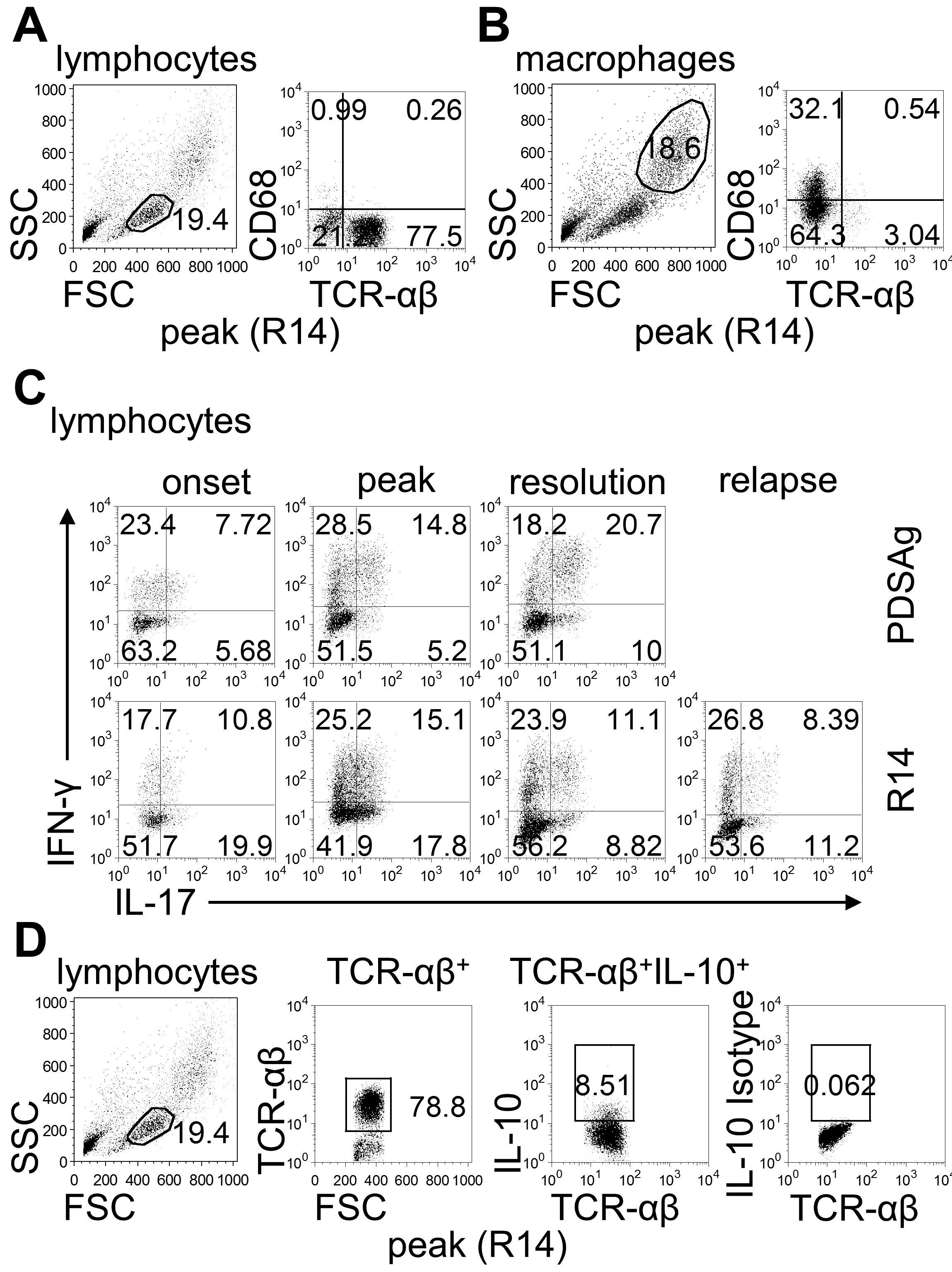

Supplement: Figure S1 — Representative dot plots from staining of intraocular cells. (A) Gating of the “lymphocyte” population of intraocular cells according to FSC and SSC (left panel) and the respective staining for TCR-αβ and monocytes/macrophages (CD68) (right panel). (B) Gating of the “macrophage” population of intraocular cells by FSC and SSC (left panel) and the respective staining for TCR-αβ and CD68 (right panel). (C) Representative dot plots of IFN-γ and IL-17 staining of the lymphocyte population, gated as shown in Fig. S1A. (D) Staining of intraocular lymphocytes (gated as shown on left panel) for TCR-αβ (versus FSC) and double staining for TCR-αβ and IL-10. The right panel shows double staining with anti-TCR-αβ and the isotype control for the anti-IL-10 antibody. (TIF) [file pone.0049008.s001.tif]

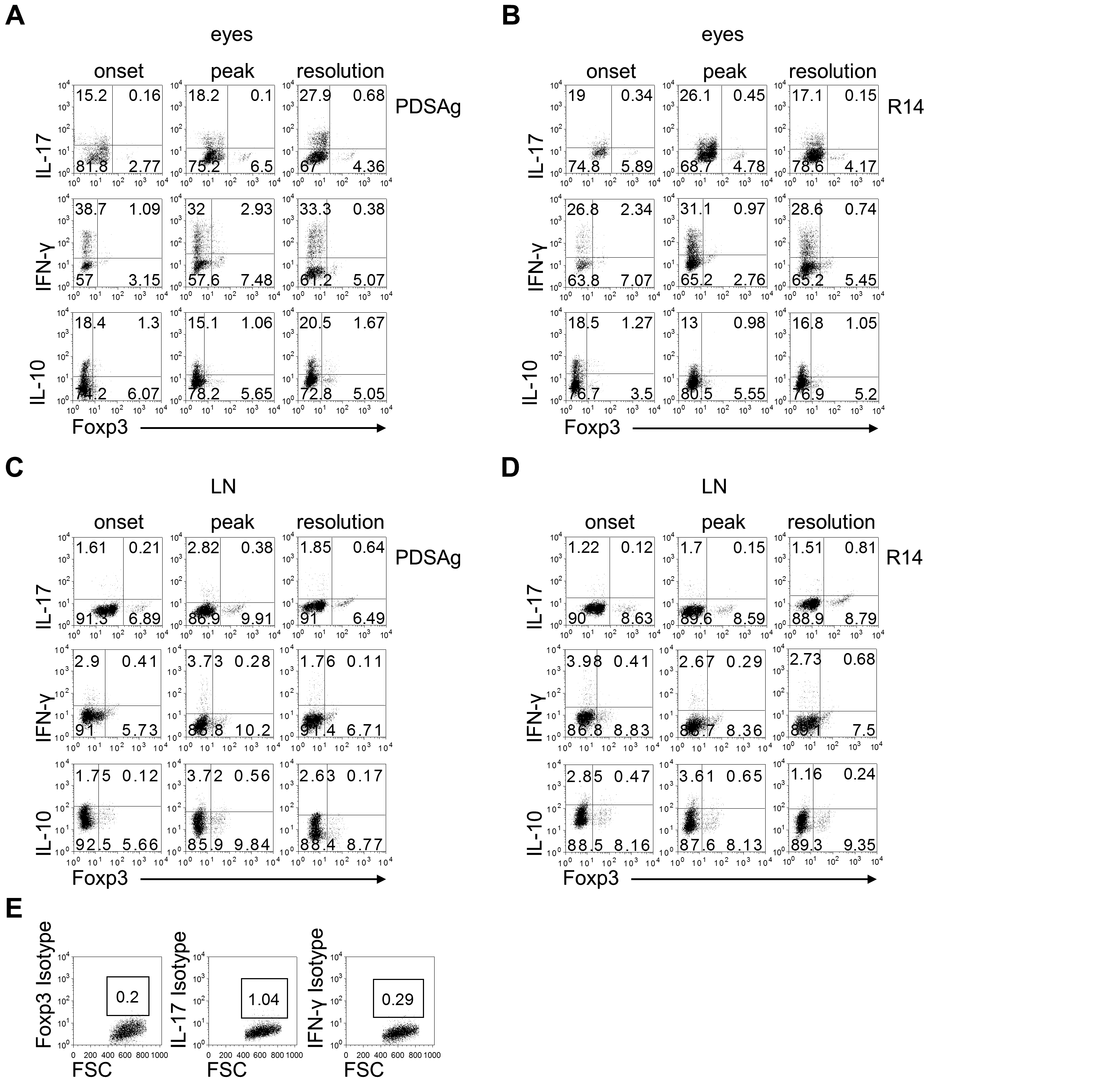

Supplement: Figure S2 — Coexpression of Foxp3 with IL-17, IFN-γ and IL-10 by intraocular and lymph node cells. Representative dot plots of the FACS analysis of “lymphocyte”-gated (see Fig. S1) intraocular (A, B) and lymph node cells (C, D) at different time points of PDSAg- and R14-CFA-induced uveitis. Cells were stained for Foxp3 and coexpression of IL-17 (upper panels), IFN-γ (middle panels) or IL-10 (lower panels). (E) Representative isotype controls for anti-Foxp3, anti-IL-17 and anti-IFN-γ, shown with lymph node cells. (TIF) [file pone.0049008.s002.tif]

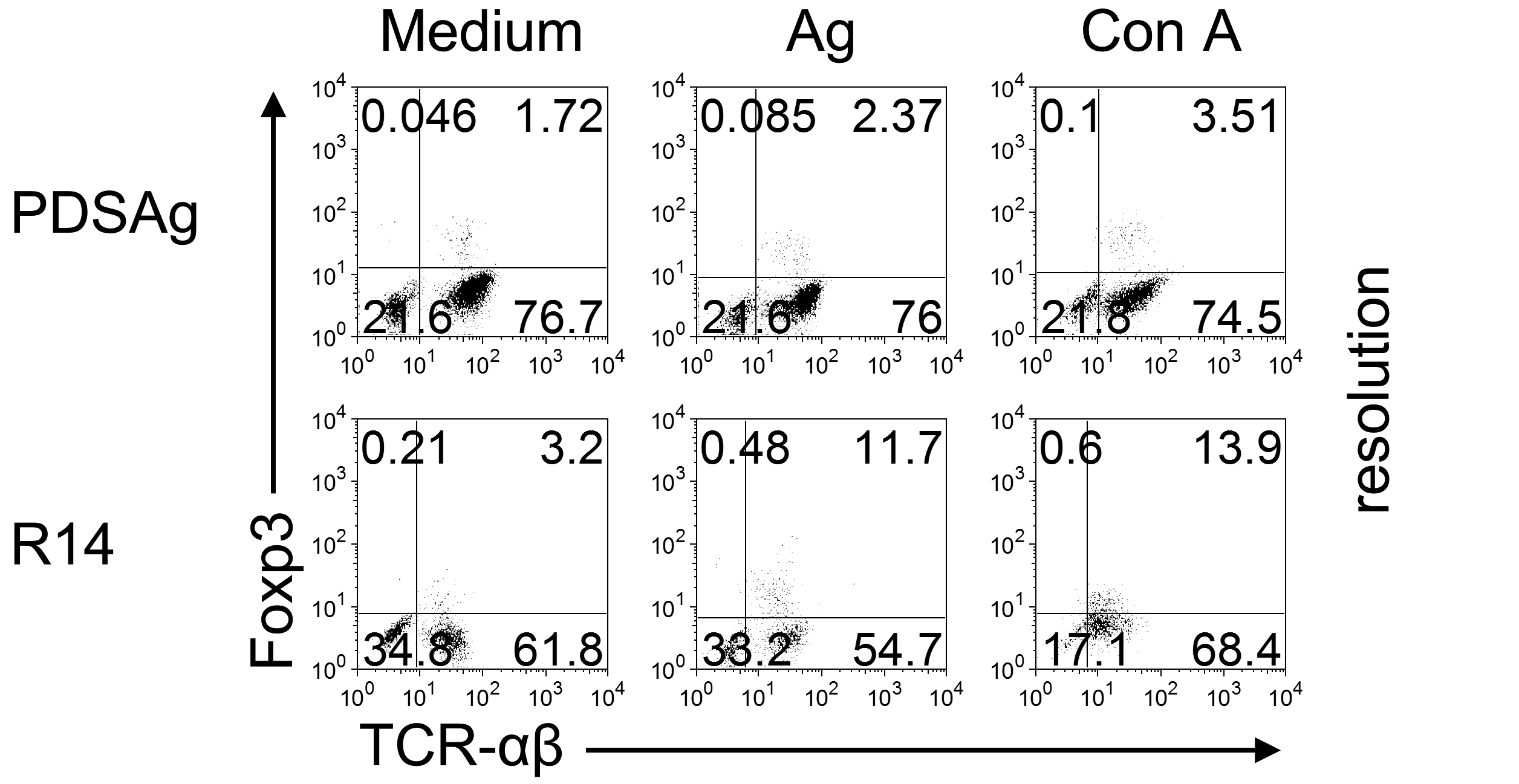

Supplement: Figure S3 — Foxp3 expression of antigen- and Con A-stimulated intraocular cells. Representative dot plots of cells from the eyes during resolution of PDSAg- and R14-induced EAU, stained for TCR-αβ and Foxp3 after 3 days of culture in medium only, specific antigen or Con A (without addition of APC). Cells were gated for lymphocytes according to SSC and FSC. (TIF) [file pone.0049008.s003.tif]
